# An adaptable, fit-for-purpose screening approach with high-throughput capability to determine speed of action and stage specificity of anti-malarial compounds

**DOI:** 10.1128/aac.00746-24

**Published:** 2024-09-12

**Authors:** Sandra Duffy, Brad E. Sleebs, Vicky M. Avery

**Affiliations:** 1 Discovery Biology, School of Environment and Science, Griffith University, Griffith, Australia; 2 The Walter and Eliza Hall Institute of Medical Research, The University of Melbourne, Parkville, Australia; 3 Department of Medical Biology, The Walter and Eliza Hall Institute of Medical Research, The University of Melbourne, Parkville, Australia; The Children's Hospital of Philadelphia, Philadelphia, Pennsylvania, USA

**Keywords:** anti-malarial agents, speed of action, *Plasmodium falciparum*, drug resistance, high-throughput screening, stage specificity

## Abstract

A revamped *in vitro* compound identification and activity profiling approach is required to meet the large unmet need for new anti-malarial drugs to combat parasite drug resistance. Although compound hit identification utilizing high-throughput screening of large compound libraries is well established, the ability to rapidly prioritize such large numbers for further development is limited. Determining the speed of action of anti-malarial drug candidates is a vital component of malaria drug discovery, which currently occurs predominantly in lead optimization and development. This is due in part to the capacity of current methods which have low throughput due to the complexity and labor intensity of the approaches. Here, we provide an adaptable screening paradigm utilizing automated high content imaging, including the development of an automated schizont maturation assay, which collectively can identify anti-malarial compounds, classify activity into fast and slow acting, and provide an indication of the parasite stage specificity, with high-throughput capability. By frontloading these critical biological parameters much earlier in the drug discovery pipeline, it has the potential to reduce lead compound attrition rates later in the development process. The capability of the approach in its alternative formats is demonstrated using three Medicines for Malaria Venture open access compound “boxes,” namely Pathogen Box (malaria set—125 compounds), Global Health Priority Box [Malaria Box 2 (80 compounds) and zoonotic neglected diseases (80 compounds)], and the Pandemic Response Box (400 compounds). From a total of 685 compounds tested, 79 were identified as having fast ring-stage-specific activity comparable to that of artemisinin and therefore of high priority for further consideration and development.

## INTRODUCTION

In 2022, there were approximately 249 million recorded cases of malaria globally and 608,000 deaths, predominantly (~462,000) in children younger than 5 years of age. Chemotherapy along with insecticidal-sprayed bed nets has proven to be the backbone for the treatment and control of malaria for many decades (1). However, resistance to most anti-malarial drugs, including those providing the basis of current frontline anti-malarial treatments, namely artemisinin combination therapies, is prevalent. After a steady decline in both malaria cases and associated deaths from 2000 to 2015, both incidence and death rate have stabilized. The reason behind this plateau is multifactorial, including overall reduced funding, resistance to anti-malarial drugs and insecticides, reduced effectiveness of diagnostics, insufficient surveillance, primary healthcare limitations while dealing with COVID-19, and changing climate (1). Thus, there is an ever increasing need to develop new anti-malarial drugs for malaria control and its eventual eradication.

With the ability to culture *Plasmodium falciparum* (*Pf*) *in vitro* continuously (2, 3), assays capable of identifying compounds with anti-malarial activity and amenable to high-throughput screening (HTS) have been developed (4–8), and numerous large compound libraries screened between 2008 and 2010 (9–11), expediting malaria drug discovery. Over the past 15 years, multiple clinical candidates have eventuated from these and other HTS screening campaigns, but most compounds identified have not been taken further in terms of extended biological evaluation. In general, there are several reasons for this, including the sheer number of compounds involved (20,000–30,000), the capabilities available for downstream hit compound triaging from both the biological and chemical perspectives, associated costs, and, to a certain extent, medicinal chemistry-led prioritization based on chemical synthesis feasibility and/or prior chemical or target-based knowledge, without further extended biological data acquisition (12). An additional concern for malaria drug discovery is that despite the broad diversity of chemistry of the hit compounds, a limited number of targets are repeatedly identified, many of which drug resistance is already prevalent (13, 14).

It is well recognized that not only fast-acting anti-malarial compounds are needed for best clinical outcomes but also the potential for a lower propensity for developing *de novo* resistance (14). However, there does exist a need for new drugs with slower mechanisms of cellular death to act as partner drugs for those which are faster acting. Hence, it is imperative that new chemical entities with diverse mechanisms of action, covering both treatment and prophylactic use, are incorporated into the therapeutic pipeline. Having an adaptable, fit-for-purpose screening approach, which not only identifies anti-malarial activity but also simultaneously provides a classification of activity based on both initial asexual speed of action and asexual blood stage specificity of growth arrest, will assist in uncovering compounds with suitable attributes for further development.

One of the first published studies to provide *in vitro* identification of speed of compound, action acknowledged as the most translatable to *in vivo* parasite clearance times (15, 16), is the parasite reduction ratio (PRR) assay. This assay involves the incubation of fixed concentrations of test compound with a defined number of asynchronous parasites, over a range of incubation times (24, 48, 72, 96, and 120 h). This method requires compound replenishment every 24 h, culture sampling, and drug removal, before the serial dilution of the drug-treated parasite sample in fresh non-infected red blood cells (RBCs). The final detection of parasites, in this case by tritiated hypoxanthine (15) or other methods of parasite detection, e.g., histidine-rich protein 2 (HRP-2) (17), allows determination of the PRR. This approach, although providing valuable and extensive information, is labor intensive with outputs taking at least 28 days. Prioritization of small numbers of compounds is therefore essential and, as such, occurs predominantly after lead identification and optimization, involving multiple iterations of chemical modifications of the initial lead compound.

To increase the number of compounds that can be evaluated at any given time, alternative approaches to determine the speed of kill have been developed. Le Manach et al. (18) employed a constant dose-response compound exposure of asynchronous parasites for 24, 48, and 72 h (IC_50_-fold shift), with tritiated hypoxanthine indirect detection of parasitemia after incubation, along with an evaluation of stage specificity by incubation of compounds against young rings or young schizonts. This approach provided a classification of compound speed of action of fast and not-fast, along with a broad indication of stage specificity. A second approach by Linares et al. (19) is similar to the PRR, detecting parasite viability by monitoring newly infected RBC after 24- and 48-h compound exposure of asynchronous parasites using fluorescently labeled non-infected RBC and the detection of newly invaded parasite with Hoechst staining by dual-fluorescence flow cytometry. Both these approaches, including others (20), have increased the capability to identify fast-acting anti-malarial active compounds but still lack published demonstration of medium- to high-throughput screening capability.

Having an adaptable, fit-for-purpose screening approach, which not only identifies anti-malarial activity but simultaneously provides a classification of activity based on both initial speed of action and parasite stage specificity, will advance early-stage drug discovery efforts for novel anti-malarial drug candidate identification.

**Fig 1:**
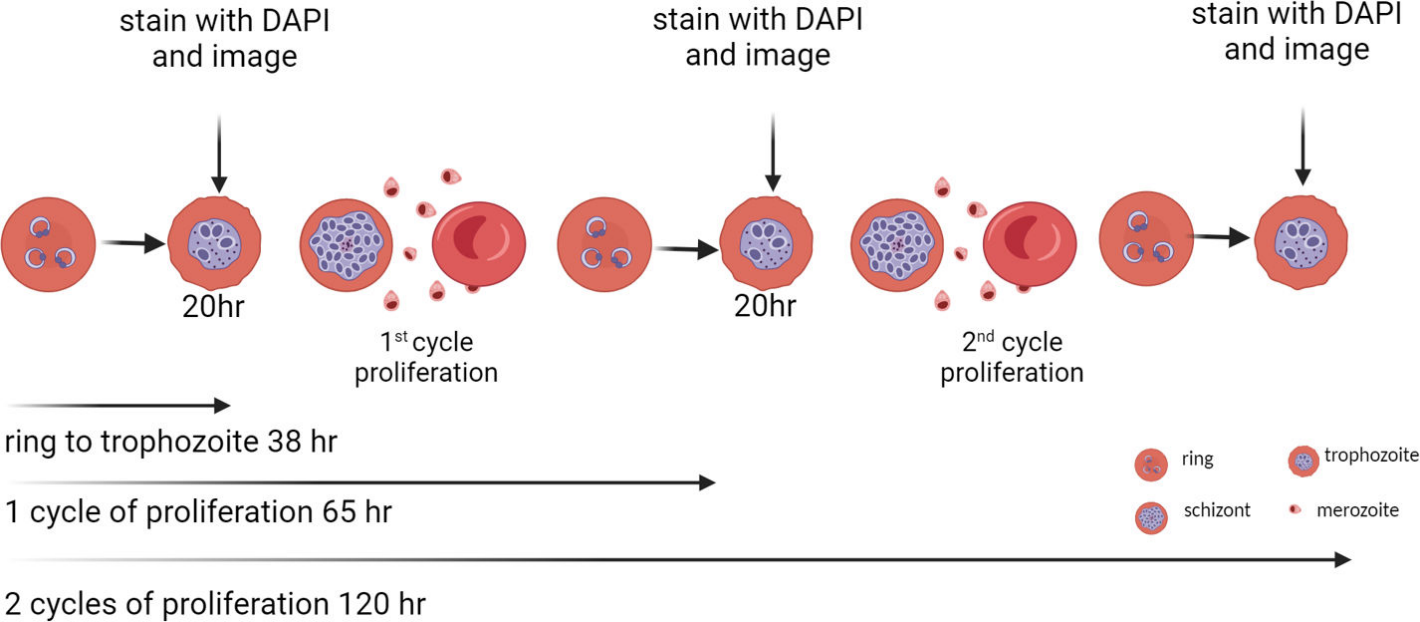
Schematic for the evaluation of anti-malarial compound speed of action and stage of parasite arrest. Highly synchronous 0–3-h ring-stage parasites were prepared using magnetic column isolation of mature schizonts and the addition of fresh non-infected RBC. After 3 h of incubation and the observed presence of new ring-stage-infected RBC, remaining schizonts were removed by sorbitol treatment, thus providing 0–3-h ring-stage parasites. Three sets of compound dose-response plates were prepared, parasites added at 2% parasitemia/0.3% hematocrit, and plates incubated (37°C, 5% O_2_, and 5% CO_2_). One plate from each of the three compound plate sets was stained with DAPI after 38 h, another after 65 h, and the third at 120 h. The plates were imaged using a Revvity Phenix imaging system, and the number of classified spots/parasites identified. All data were normalized as percent inhibition of either parasite development (38 h SMIA) or prevention of proliferation (65 and 120 h) in comparison to 5 µM puromycin and 0.4% dimethyl sulfoxide (DMSO) controls. The IC_50_ value for each compound was determined using a nonlinear sigmoidal dose-response calculation with no constraints on either the top or bottom of the generated curves. The Emax (activity plateau) and IC_50_ values were tabulated for each test compound at each of the three time points. This figure was generated using Biorender.

The screening approach described here is adaptable to accommodate the various aspects of early-stage anti-malarial drug discovery and is based on our highly validated confocal imaging assay (21), which quantifies parasite number utilizing DAPI (4′,6-diamidino-2-phenylindole) DNA staining. This confocal imaging assay has been utilized to screen >5 million compounds, including the AstraZeneca corporate collection (500,000 compounds) (22), the Evotec commercial library (>250,000 compounds) (23), and smaller compound diversity libraries [20,000 Takeda pharmaceuticals (24) or target-based (25, 26) compound libraries], demonstrating validated capabilities and capacity in early-stage malaria drug discovery. To further extend the versatility and reach of this assay to incorporate determination of speed of action and parasite stage specificity of parasite growth arrest, it was adapted to identify compound effects on the development of the parasite from ring to early schizont stage, i.e., comparable to the World Health Organization schizont maturation assay (27). The development of this automated imaging-based schizont maturation inhibition assay (SMIA) involved two adaptations to the original HTS assay. First, highly synchronous 0–3 h post RBC invasion, ring-stage parasites were used (in all evaluations), and parasite fluorescent staining with DAPI was performed at 38 h post exposure to the test compounds, i.e., at mature trophozoite/early schizont stage before parasite proliferation occurs. To ensure direct comparison between the SMIA and time points, which represent one complete cycle of replication with detection at early trophozoite (65 h) or after two cycles of proliferation (120 h) and development to early trophozoite, compound testing was performed on the same culture preparation, and identical exposure parameters and image analysis script parameters were utilized. The screening approach described herein uses the IC_50_-fold shift evaluation as demonstrated by Le Manach et al. (18), with parasite viability determined based on parasite proliferation as previously used by Linares et al. (19). The compound testing approach is presented in Fig. 1.

To validate the screening approach for identifying anti-malarial activity and simultaneous classification of the initial speed of action and stage of parasite arrest, a set of well-documented anti-malarial drugs were evaluated, combined with 125 anti-malarial (28) compounds from the Medicines for Malaria Venture (MMV) Pathogen Box were tested. Subsequently, 160 compounds from the Global Health Priority Box [GHPB; zoonotic neglected diseases (ZND; 80 compounds) and Malaria Box 2 (MB2; 80 compounds) sets] and 400 from the Pandemic Response Box were also tested in an abridged version, where two compound doses (10 and 5 µM) at two time points (38 and 120 h) were employed to prioritize fast ring-stage active compound selection in higher throughput. Those designated as potentially fast acting and a small selection of slow-acting compounds were then evaluated using the full IC_50_-fold shift approach (Fig. 1) to confirm the speed of action and stage specificity classification. Puromycin, the nonspecific protein synthesis inhibitor active at all stages of *Pf* asexual development (29), was used as the positive 100% inhibition control in conjunction with 0.4% DMSO (negative control) to normalize all data for percent inhibition calculation.

## RESULTS

### Development of the schizont maturation inhibition assay

*Pf* asexual blood stage development from ring stage to trophozoite and, finally, schizont involves DNA replication. We hypothesized that *Pf* development stages could be distinguished by measuring DNA content using DAPI staining, as used in the original HTS assay (21). To test this possibility, the SMIA was developed on the foundations of the HTS asexual imaging assay (21). The change in parasite detection throughout development from ring to schizont was evaluated as described in Materials and Methods.

**Fig 2:**
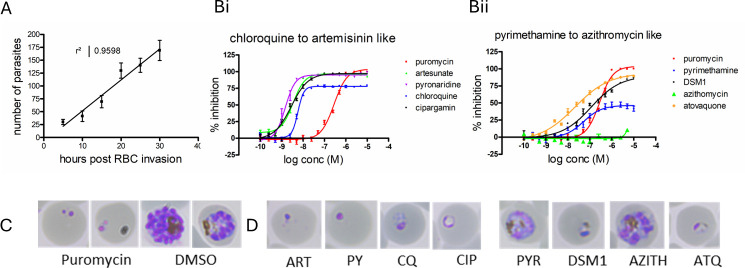
Development and validation of the schizont maturation inhibition assay. (A) Demonstration of stage effect determination of *Pf*3D7 over time based on parasite detection in relation to parasite size and fluorescent intensity. Hours post RBC invasion plotted against the number of classified parasites. (B) Reference compound IC_50_ determination in the SMIA. (Bi) Reference compounds with fast speed of action, puromycin, artesunate (ART), chloroquine (CQ), pyronaridine (PY), and cipargamin (CIP). (Bii) Reference compounds with a slow or delayed death speed of action as proposed by scientific literature. DSM1, pyrimethamine (PYR), atovaquone (ATQ), and azithromycin (AZITH) with puromycin for comparison. Data are the mean ± SD for three biological replicates performed in duplicate point, i.e., six data points per concentration from three independent biological replicates. The top plateau of each curve (Emax) represents the stage/age of parasite at which the parasite population arrested growth at 38 h post RBC invasion. This is demonstrated by pyrimethamine which has a low Emax of approximately 40% in comparison with puromycin which has a 100% Emax. (C) Giemsa-stained images of puromycin and DMSO controls at 38 h. (D) Representative Giemsa smears of reference compound exposed *Pf*3D7 after 38 h of incubation.

Fortuitously, the identical image analysis script used for the HTS assay in its current format, utilizing the Phenix High Content Imaging System, was discriminated between ring stages and their transition to more mature forms based on both size and intensity of DNA-stained objects (Fig. 2A). As expected, the number of classified spots increased with time post RBC invasion, representing the changing nucleic acid quantity and distribution within the developing parasite over time. Time points prior to 5 h and beyond 38 h of parasite development were not linear (data not shown). However, these data indicated strongly that the use of highly synchronous parasites and the identical imaging parameters and analysis script used in a 72-h proliferation assay should enable the identification of compounds in preventing the parasite development from ring stage to mature trophozoite/early schizont stage. The number of parasites identified at time points post RBC invasion is presented in Fig. 2A.

### Validation of the SMIA to classify reference anti-malarial drugs as fast or slow acting, with indication of parasite stage specificity

Nine anti-malarial drugs with well-validated mechanisms of action and/or speed of action data publicly available were evaluated using the SMIA. These included artesunate (ART), chloroquine (CQ), pyrimethamine (PYR), pyronaridine, cipargamin, DSM1, atovaquone, azithromycin, and puromycin. In all evaluations, puromycin was used as 100% inhibition at 5 µM and 0.4% DMSO as the negative control, which were used to normalize the data to percent inhibition for determination of compound IC_50_ values (Fig. 2Bi and Bii).

Puromycin, artesunate, pyronaridine, and cipargamin were classified as fast acting against early and more mature parasite ring stages in the case of chloroquine. Cipargamin has a very distinct morphological phenotype (Fig. 2D, CIP) where the central portion of the parasite was expanded, DNA located at the edges of the parasite, and no hemozoin is present. Due to the low levels of DNA present in the cipargamin-treated parasites, the automated analysis script identified these parasites as having a >90% Emax and therefore active against ring-stage parasites.

Both DSM1 and atovaquone were characterized as fast ring-stage active compounds in the SMIA, whereas their activity is well established to be slow with >48 h of lag phase (15). Both compounds had a very low Hillslope in comparison to puromycin, which may indicate a concentration-dependent slow mechanism of action. When tested in the full 38-, 65-, and 120-h evaluation (Fig. 1), these compounds were identified to be slow acting based on IC_50_-fold shift.

### Reference data set for SMIA 1 and 2 cycles of proliferation

**Fig 3:**
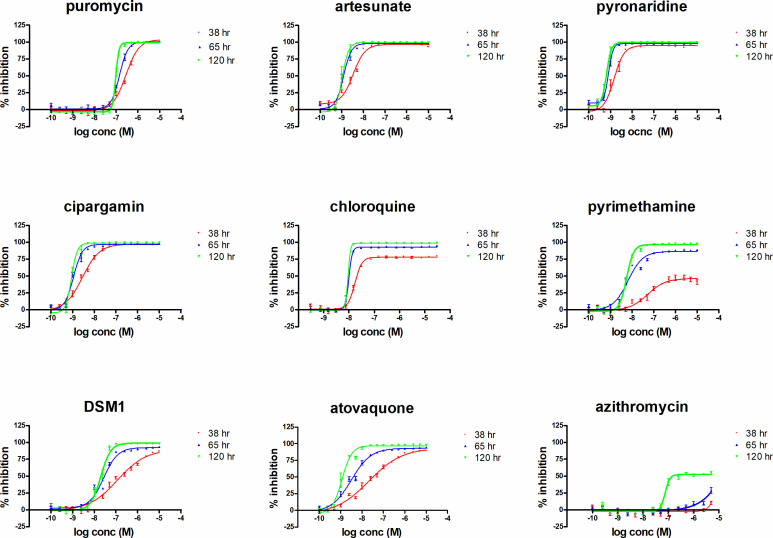
Reference compound speed of action and parasite stage of arrest profiles. IC_50_ plots for reference compounds artesunate, chloroquine, pyrimethamine, pyronaridine, cipargamin, DSM1, atovaquone, azithromycin, and puromycin at 38, 65, and 120 h. The data points represent the mean and standard deviation of *n* = 3 biological replicates performed in duplicate point for 38 and 65 h and *n* = 2 biological replicates for 120 h.

The reference compound test plates for artesunate, chloroquine, pyrimethamine, pyronaridine, cipargamin, DSM1, atovaquone, azithromycin, and puromycin were prepared as described for the SMIA evaluation (described in Materials and Methods), with the exception that three replicates of each assay plate were prepared and incubated together. Thus, all testing was against the identical parasite culture for all three time points for direct comparison. After 38 h, one plate was removed from incubation and stained with DAPI, followed by the second and third plate after 65 and 120 h of incubation, respectively. The data were normalized against the puromycin and 0.4% DMSO controls, and IC_50_ values were determined, as outlined for the SMIA evaluation. Data are presented in Fig. 3.

As observed in Fig. 2, both DSM1 and atovaquone data for the SMIA (38 h) resulted in a shallow hill slope (red line), which at later time points (blue and green) became more comparable with the other compounds tested. The data obtained for the reference compounds tested at the three time points are presented in Table 1.

**TABLE 1:** Reference compound activity data^*a*^

Compound ID	SMIA 38 h	38 h Emax at plateau	65 h	Fold change IC_50_ 38/65 h	120 h	Speed of action classification
	IC_50_ nM ± SD	% Inhibition	IC_50_ nM ± SD		IC_50_ nM ± SD	
Artesunate	3.07 ± 0.22	96.7	1.4 ± 0.13	2.17	1.6 ± 0.41	Fast
Cipargamin	2.81 ± 0.27	97.3	1.3 ± 0.12	2.17	1.2 ± 0.07	Fast
Puromycin	218 ± 30.3	103	142 ± 19.6	1.53	95.4 ± 2.79	Fast
Pyronaridine	1.47 ± 0.79	95.1	0.81 ± 0.21	1.81	0.66 ± 0.15	Fast
Chloroquine	17.2 ± 2.46	77.9	10.9 ± 1.39	1.57	10.1 ± 0.72	Intermediate
Pyrimethamine	51.3 ± 12.5	46.5	6.4 ± 1.24	8	5.6 ± 0.58	Slow
DSM1	116.8 ± 80.2	89.8	27.2 ± 6.12	4.29	19.8 ± 1.39	Slow
Atovaquone	21.0 ± 10.9	93.9	3.1 ± 1.50	6.68	1.2 ± 0.24	Slow
Azithromycin					75.5 ± 0.47	Delayed death

^*a*^ IC_50_ values at each time point, the SMIA (38 h) Emax, and IC_50_-fold change values between 38 and 65 h of incubation. The speed of action classification was determined as follows: >90% Emax and IC_50_ -fold shift <2.5 (artesunate, cipargamin, puromycin, and pyronaridine) were designated as fast ring stage and between 70% and 89% with an IC_50_-fold shift of <2.5 designated as intermediate (chloroquine). Those with IC_50_-fold changes greater than 2.5 were designated as slow-acting compounds (pyrimethamine, DSM1, and atovaquone). The slow-acting compounds were further classified based on the Emax plateau of between 37% and 70%, indicating parasite arrest at trophozoite, e.g., pyrimethamine. Azithromycin was classified as having a delayed death mechanism. Data are from three biological replicates in duplicate point for the SMIA 38 and 65 h, and two biological replicates (duplicate point) for the 120-h data.

The reference compounds each demonstrated distinctly different profiles depending on parasite stage of arrest (38 h Emax and IC_50_-fold change; Table 1). Puromycin, artesunate, pyronaridine, and cipargamin demonstrate a fast speed of action, causing parasite arrest at young ring stages (Emax >90% inhibition). These compounds were therefore considered to be fast acting with activity against ring-stage parasites as previously reported (29–33). Chloroquine had a reduced Emax (77.9%) in comparison to puromycin, artesunate, pyronaridine, and cipargamin, indicating an effect on slightly more mature parasite stages as expected due to its effect on hemozoin formation and digestive vacuole interactions (34). Pyrimethamine demonstrated parasite arrest at the trophozoite stage (as observed by Giemsa smear evaluation at 38 h of incubation and an Emax of 51%), where activity increased after the first cycle of proliferation (eightfold increase) and thus a slower speed of action than those considered to be fast acting. This activity profile was consistent with DHFR (dihydrofolate reductase) inhibitors (18). Atovaquone and DSM1 have unique activity profiles, causing a delay in parasite development which initially appeared as activity against young ring stages but was, in this context, considered a lag phase represented by the fold increase in activity with parasite proliferation into the next development cycle, as observed by Le Manach et al. (18). Azithromycin had a well-recognized second-generation/delayed death phenotype (35), which, in this evaluation activity, was only observed to reach a plateau within the second cycle of proliferation, as expected.

Based on this provisional evaluation, 125 compounds from the MMV Pathogen Box were evaluated in the SMIA, and both single and double proliferation cycles in IC_50_ dose-response format.

### MMV Pathogen Box malaria

The 125 compounds of the MMV Pathogen Box designated the malaria set were evaluated for speed of action and stage of arrest using the format described in for the reference compounds. A total of 15 × 384 well plates per incubation time point were processed for this evaluation. Each plate contained 16 wells of 0.4% DMSO and 16 of 5 µM puromycin controls. An average *Z*′ value of 0.71 ± 0.02 was obtained for the SMIA (38 h), demonstrating very good assay performance. The Z′ values for 65- and 120-h evaluations were all greater than 0.68 as expected for this well-validated assay. The data for all compounds, in each timed assay, were normalized to 0.4% DMSO and 5 µM puromycin to give percent inhibition, and the data plotted to provide IC_50_ values.

Compounds with insufficient activity to generate an IC_50_ value at any of the three time points were excluded from the data set for further analysis. The fold change in IC_50_ values between 38 and 65 h, and 38 and 120 h was also calculated for each compound.

The data obtained from this complete evaluation classified compound activities into fast ring stage [artemisinin-like SMIA (38 h) Emax >90%, IC_50_ shift <2.5], intermediate [chloroquine-like SMIA (38 h) Emax 70%–89%], slower trophozoite stage [pyrimethamine-like SMIA (38 h) Emax 37%–70%), 48-h lag phase (atovaquone-like Emax >80% IC_50_ shift >2.5), those involving either egress or invasion [IC_50_ unobtainable in SMIA (38 h) as parasite stage of arrest was later than 38 h), and those demonstrating activity only in the second generation (azithromycin like). Of the 95 compounds for which an IC_50_ value was obtained for at least one of the incubation periods, 28 were classified as fast ring stage (artemisinin like; Table 2), 11 comparable to chloroquine, 27 comparable to pyrimethamine, 8 identified as atovaquone like, 16 with activity against late-stage trophozoites/schizonts or inhibitors of invasion, and 2 which demonstrated a delayed death phenotype comparable to azithromycin. The complete data set for all 95 compounds is provided in Table S1. Each classification set is discussed below.

**TABLE 2:** Compounds identified from the MMV Pathogen Box malaria compound set with artemisinin-like speed of action^*f*^

	This study								Tougan et al. (36)	Dans et al. (37)	Dans et al. (37)	Patra et al. (38)	Barnes et al. (39)
Compound ID	SMIA % inhibition (Emax)	SMIA IC_50_ (µM)	65-h Ring (Emax)	65-h Ring IC_50_ (µM)	120 h (Emax)	120-h Ring IC_50_ (µM)	38/65 h Ratio	Literature target/speed of action/stage of arrest			% Inhibition ring growth		
MMV010764	101	1.05 (0.909–1.20)	102	1.05 (0.903–1.22)	100	1.48 (1.00–2.18)	1.0	INE936 analog	PYR like				
MMV024035	93	0.333 (0.289–0.384)	97	0.33 (0.261–0.420)	99	0.40 (0.346–0.452)	1.0		Ring-CQ like	Invasion	100		
MMV676380	99	2.51 (0.849–0.740)	100	2.41 (1.87–3.09)	99	2.00 (1.62–2.47)	1.0	DV^*a*^	Ring-CQ like				
MMV407834	102	2.19 (1.95–2.46)	104	2.04 (1.58–2.64)	100	3.35 (2.79–4.01)	1.1	Early trophozoite	Troph/schizont				
MMV022478	93	2.15 (1.93–2.41)	97	1.95 (1.76–2.15)	100	3.08 (2.15–1.42)	1.1	CPDK?	Ring-CQ like				
MMV020391	99	2.65 (2.07–3.38)	96	2.40 (2.14–2.70)	97	2.70 (1.73–4.21)	1.1	ATP4^*b*^	ART like	Invasion	IA	Egress	
MMV667494	97	0.104 (0.095–0.114)	99	0.09 (0.087–0.100)	99	0.08 (0.072–0.093)	1.1	*Pf*eEF2*^c^/PfIVT^d^*	ART like	Invasion/egress	100		
MMV022029	98	0.733 (1.24–1.16)	83	0.64 (0.588–0.826)	96	0.88 (0.743–1.051)	1.1		Ring-CQ like				
MMV032967	98	1.74 (1.34–2.26)	94	1.52 (1.21–1.91)	98	2.00 (na)	1.1	DV^*a*^	Ring-CQ like				
MMV020136	95	1.61 (1.45–1.79)	93	1.38 (1.10–1.73)	97	1.46 (1.31–1.63)	1.2	ATP4^*b*^	ART like	Invasion	10.7		ATP4-egress
MMV020623	103	3.34 (2.71–4.06)	99	2.74 (2.25–3.55)	99	3.96 (na)	1.2	ATP4^*b*^	ART like	Invasion	29	Egress	
MMV020710	99	0.633 (0.543–0.739)	96	0.47 ((0.422–0.527)	98	0.60 (0.553–0.660)	1.3	ATP4^*b*^	ART like	Invasion	24.2	Egress	ATP4-egress
MMV020081	99	0.511 (0.428–0.612)	96	0.35 (0.307–0.406)	97	0.45 (0.356–0.556)	1.4	ATP4^*b*^	ART like	Invasion/egress	37.9	Egress	
MMV023233	96	0.11 (0.090–0.134)	98	0.08 (0.064–0.090)	97	0.10 (0.090–0.103)	1.5		Ring-CQ like	Invasion	100		
MMV020165	93	1.69 (1.30–2.17)	101	1.12 (0.613–2.05)	97	1.50 (1.38–1.63)	1.5		PYR like				
MMV634140	102	0.341 (0.289–0.401)	99	0.220 (0.175–0.273)	97	0.170 (0.158–0.187)	1.6	PfeEF2^*c*^/PfIVT	ART like	Invasion	83.2		
MMV006239	102	2.95 (2.28–3.81)	95	1.87 (0.667–4.85)	98	2.23 (1.93–2.57)	1.6	ATP4^*b*^	ART like	Invasion/egress	18.3	Egress	ATP4-egress
MMV019721	102	2.2 (1.98–2.45)	100	1.39 (1.28–1.52)	100	1.63 (0.689–3.87)	1.6	Acetyl-CoA synthetase^*d*^	ART like	Invasion	46.5		
MMV000907	108	3.03 (2.37–3.88)	110	1.91 (1.34–2.72)	94	1.22 (1.01–1.49)	1.6		PYR like				
MMV062221	106	4.25 (3.35–5.39)	101	2.63 (2.19–3.17)	99	2.76 (2.28–3.34)	1.6		IA				
MMV393144	92	1.8 (1.56–2.08)	88	1.00 (0.913–1.10)	95	0.98 (0.914–1.04)	1.8		PYR like				
MMV085071	98	0.191 (0.172–0.211)	98	0.100 (0.092–0.117)	99	0.120 (0.104–0.129)	1.8	DV^*a*^	PYR like			Egress	
MMV023985	92	5.27 (4.41–6.30)	90	2.84 (2.18–3.71)	95	2.43 (0.846–6.99)	1.9		Troph/schizont				
MMV024443	101	3.57 (3.05–4.18)	95	1.84 (1.29–2.62)	98	1.65 (1.48–1.84)	1.9	PfCDPK1^*e*^	Troph/schizont			Egress	
MMV019551	108	1.81 (1.20–2.71)	97	0.93 (0.70–1.30)	95	0.80 (0.701–0.910)	2.0		ART like				
MMV020670	98	3.52 (3.03–4.10)	93	1.74 (1.47–2.07)	96	1.94 (na)	2.0		PYR like			Egress	
MMV007803	103	3.17 (2.52–3.99)	99	1.54 (1.15–2.07)	94	1.90 (1.99–1.78)	2.1		PYR like				
MMV020120	102	1.47 (1.17–1.85)	95	0.66 (0.517–0.839)	96	0.75 (0.661–0.582)	2.2		ART like				

^*a*^ Reference (40).

^*b*^ Reference (41).

^*c*^ Reference (42).

^*d*^ Reference (43).

^*e*^ Reference (44).

^*f*^ Activity data from this study are compared with that from four other studies and indications of target, speed of effect, stage of arrest, and if they are analogs of compounds with associated known drug targets, speed of action, and/or stage of arrest. Data are from a single biological replicate performed as two technical replicates. IC_50_ values are the average of the two technical replicates, and the 95% confidence interval values are provided within brackets. na = no confidence interval provided by Prizm software. IA, inactive. DV, digestive vacuole. CPDK, calcium-dependent protein kinase.

#### Artemisinin like

The proportion of fast ring-stage active compounds (28/95, 29%) could be considered high based on previous observations that older anti-malarials are thought to have their action against trophozoites (45). However, within these 28 compounds, 6 were proposed to work through ATP4 (33, 41), 2 PfeEF2/*PfIVT* (42), 1 acetyl-CoA synthetase (ACS) inhibitor (43), 1 compound is an analog of INE963 recognized as having a speed of action comparable with artemisinin (46), and 3 compounds have effects on the digestive vacuole and are potentially programmed cell death modulators (40). Therefore, 12/28 (42%) compounds have proposed mechanisms of action associated with fast ring-stage activity.

Tougan et al. (36), using flow cytometry, evaluated the stage of effect of the MMV Pathogen Box malaria compound set (125 compounds). Overall, the study by Tougan et al. (36) had a 55% correlation for fast ring-stage actives with this study (Table S1). The 125 Pathogen Box malaria compound set has also been evaluated for inhibitors of parasite egress and invasion by two separate approaches in which the outcomes did not show any correlation with each other. Patra et al. (38) identified predominantly inhibitors of egress, while Dans et al. (37) identified predominantly inhibitors of invasion and those which appeared to have effects on both egress and invasion. On more detailed investigation, Dans et al. (37) identified several compounds as inhibitors of ring-stage-specific development (>35% inhibition at 2 µM, 4 h of compound exposure), including the two PfeEF2 compounds [MMV667494 (100%) and MMV634140 (83.2%)], the ACS compound [MMV019721 (46.5%)], a single ATP4 compound [MMV020081 (37.9%)], and two compounds with unknown targets [MMV024035 (100%) and MMV023233 (100%)]. These six compounds were also identified as having activity on ring stages by Tougan et al. (36) using flow cytometry.

Dennis et al. (41) identified 11 of the 125 compounds as having an ATP4-like phenotype. Of the 11 compounds, in this study, 6 (MMV020391, MMV020136, MMV020623, MMV020710, MMV020081, and MMV006239) were classified as fast ring-stage artemisinin like, 3 (MMV001059, MMV000858, and MMV085210) as intermediate chloroquine like, and 1 (MMV020520) in the egress/invasion group, plus 1 compound which was not active in this study. This spread of speed of activity for proposed ATP4 active compounds has been recorded previously by Ullah et al. (20), which was indicated to be dependent on chemotype. Of the ATP4 proposed compounds, three were further studied by Barnes et al. (39), who identified the activity of these compounds (MMV020136, MMV020710, and MMV006239) as inhibitors of schizont egress. Although between studies on invasion and egress, ATP4 active compounds demonstrated conflicting data, and in this study, on speed of action and stage specificity, the six compounds within the artemisinin-like classification with ATP4 activity attributed to them (MMV020391, MMV020136, MMV020623, MMV020710, MMV020081, and MMV006239) all demonstrate a fast activity which is supported by both *in vivo* (47, 48) and *in vitro* studies, indicating fast ring-stage activity for ATP4 targeting compounds (33, 49). MMV024035, MMV022029, MMV023233, MMV020165, MMV000907, MMV062221, MMV393144, MMV023985, MMV019551, MMV020670, MMV007803, and MMV020120 were all identified as being fast ring-stage active with no mechanism of action currently assigned. These 12 compounds are therefore of high priority for further studies to identify the associated novel high value targets.

#### Chloroquine like

Eleven compounds were classified as chloroquine like, and of these, three (MMV001059, MMV000858, and MMV085210) have previously been proposed to target ATP4 (41). The remaining eight compounds (MMV560185, MMV006372, MMV023949, MMV032995, MMV026468, MMV020388, MMV020517, and MMV007471) have no published mechanism of action.

#### Pyrimethamine like

This is the second largest group of compounds, with little published data for the majority. MMV020512 was identified as inhibiting parasite invasion by Dans et al. (37), and very recently, Dans et al. (50) also demonstrated the inhibition by MMV006833, an aryl amino acetamide, on the lipid transfer protein PfSTART1. Apart from MMV020591, with activity predicted to be through DHODH (51), the remaining 25 compounds (MMV026313, MMV023183, MMV676350, MMV024937, MMV031011, MMV084603, MMV084864, MMV006901, MMV023953, MMV020982, MMV392832, MMV024114, MMV011511, MMV021375, MMV006741, MMV023370, MMV024829, MMV023227, MMV020259, MMV676528, MMV085230, MMV020321, MMV028694, MMV007625, and MMV026490) have no published mechanism of action.

#### Atovaquone like (slow)

Of the eight compounds that demonstrated an atovaquone-like profile, two are PI4K inhibitors (MMV010576 and MMV02410) (52, 53), which based on other inhibitors of PI4K, such as MMV390048, are expected to demonstrate activities comparable to atovaquone (54). MMV010545, a CDPK inhibitor (18), and MMV021057 (azoxystrobin) have been demonstrated to act on the mitochondrial electron transport chain (ETC) of *Plasmodium* (55) both demonstrating a >2.5 fold change in IC_50_ between 38 and 65 h in this study. The remaining four compounds (MMV026356, MMV023860, MMV019234, and MMV026020) have unknown targets.

#### Egress/invasion (action after 38 h post RBC invasion-slow)

Sixteen compounds fulfilled the egress/invasion classification, with MMV020537, MMV011229, and MMV020289 being associated with DHODH (51), MMV020520 (ATP4) (37), MMV024397 (ETC) (55), and MMV030734, an analog of MMV030084 predicted to act via PKG (cGMP-dependent protein kinase) (56). The activity of MMV019087 has been investigated further by Bailey et al. (57) and is indicated to be moderate to slow acting, with activity against trophozoite stages after the first cycle of proliferation by tritiated hypoxanthine determination but not by microscopy. A second compound MMV008439 [an analog of MMV396797 and likely PI4K inhibitor (58)] also demonstrated this unusual activity profile in this study. The remaining five compounds (MMV007638, MMV011765, MMV016136, MMV024195, and MMV1030799) have no published proposed mechanism of action.

#### Delayed death—second generation

MMV020291 and MMV676605 both demonstrated a delayed death phenotype. MMV020291 has been further studied for its mechanism of action (59) and was found to act through interference with actin-1/profilin and a partial delayed death phenotype due to malformation of the apicoplast. MMV676605 (linsitinib), with an unknown mechanism of action in *Plasmodium falciparum,* is currently in clinical trials for the treatment of various cancers as an IGF-1R (tyrosine kinase) inhibitor (60). The significance of this observation is not known but may suggest that kinase inhibitors are incredibly diverse in their actions and may also result in delayed death activities.

The correlation between the proposed targets and published speed of action and stage specificity of the test compounds within the Pathogen Box 125 malaria disease set indicated that this approach distinguished fast ring-stage active compounds (Tables 1 and 2) from those with slower mechanisms. Simultaneously, the assay could be used to identify potentially unique activity profiles, such as that for MMV019087, and delayed death phenotypes, such as observed for MMV020291.

### Evaluation of an abridged two-dose, two-time point screening format

To increase the throughput of this approach, we investigated whether an abridged screening approach utilizing two moderately high screening concentrations, e.g., 5 and 10 µM, could be used to simulate a pseudo Emax-based percent inhibition at 38 h (SMIA) and 120 h in a bid to separate fast active compounds from those with a slower activity profile.

**Fig 4:**
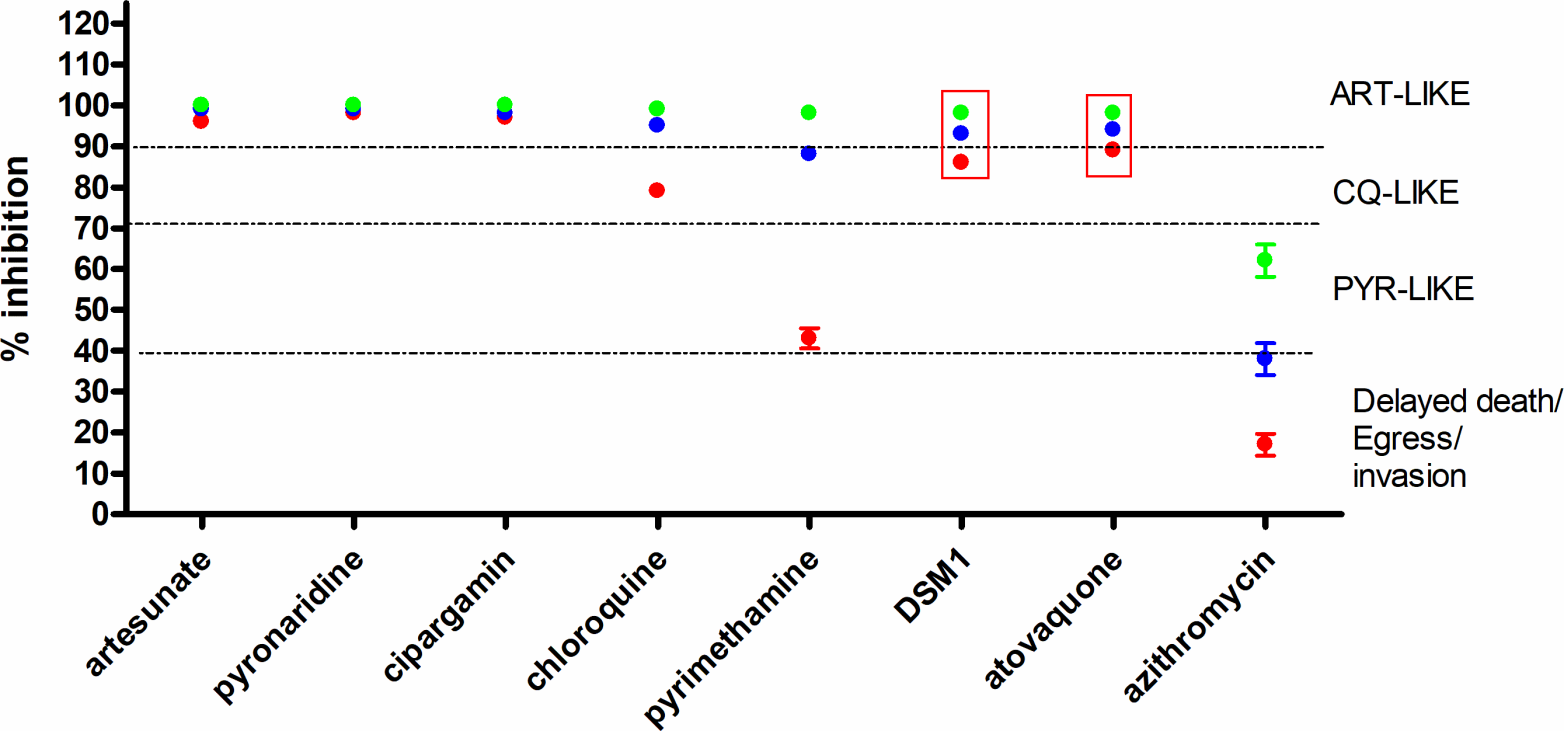
Abridged theoretical compound screening approach for high-throughput screening. Identification of fast-acting compounds using 5 and 10 µM concentrations at two time points, 38 h SMIA and 120 h. Each data point was the mean plus standard deviation of 12 (8 for 120 h) data points for each incubation time [red SMIA (38 h), green 120 h]. The 12 (or 8) data points for each incubation included those for both 5 and 10 µM generated from three (or two) separate biological replicates. The standard deviation for each data point, as demonstrated by the error bars in the graph, indicates the deviation from an average two-dose-simulated Emax value. DSM1 and cipargamin (data enclosed in red box) demonstrated a very slow mechanism of action, not always distinguishable from intermediate- to fast-acting compounds with this format.

The reference compound data (Fig. 3) were thus reanalyzed as follows and are presented in Fig. 4. The mean and standard deviation were calculated for the 10- and 5-µM percent inhibition data from the *n* = 3 (or *n* = 2, 120 h) assay data points. Compounds with SMIA percent inhibition >90% were considered as fast (artesunate like) acting. Between 70% and 90% were intermediates, i.e., chloroquine like, and those with activity between 40% and 70% were considered as slow and thus pyrimethamine like. Compounds with a percent inhibition less than 40% were the slowest working through egress or invasion inhibition, delayed death, or of insufficient activity/inactive. The 120-h data confirm the compounds were active or inactive and allow for the identification of compounds with delayed death phenotypes, such as azithromycin.

To test the ability of using two high concentrations to generate a pseudo Emax plateau in both the SMIA (38 h) and two proliferation cycles (120 h), the Global Health Priority Box and Pandemic Response Boxes were evaluated. The Global Health Priority Box contains compounds with anti-malarial activity [MB2 (“irresistibles”) and ZND], whereas the Pandemic Response Box can be considered predominantly as an anti-malarial-naïve compound library with activities and applications in other infectious diseases, including anti-bacterial, anti-fungal, and anti-viral.

### Screening the MMV Global Priority Box compounds (160 compounds) and Pandemic Response Box (400 compounds) for fast ring-stage active compounds

The compounds were tested at 5 and 10 µM in both the SMIA (38 h) and 120 h assay, in duplicate point (total four data points per assay incubation time). Assays were performed as described for the Pathogen Box evaluation. The average and standard deviation of the normalized percent inhibition data for each compound [duplicate point for two concentrations 10 and 5 µM (i.e., four data points)] were calculated for each assay time point. As numerous compounds were observed to have insolubility issues in the intermediate water dilution step [100 µM in 4% DMSO (10 µM final concentration) and 50 µM in 4% DMSO (5 µM final concentration)], compounds with activity but standard deviations >20% for either assay were separated into a subgroup to avoid misclassification of activities (21 compounds from ZND and 20 from MB2).

### Primary screening of the GHPB and Pandemic Response Box

#### Zoonotic neglected disease

**Fig 5:**
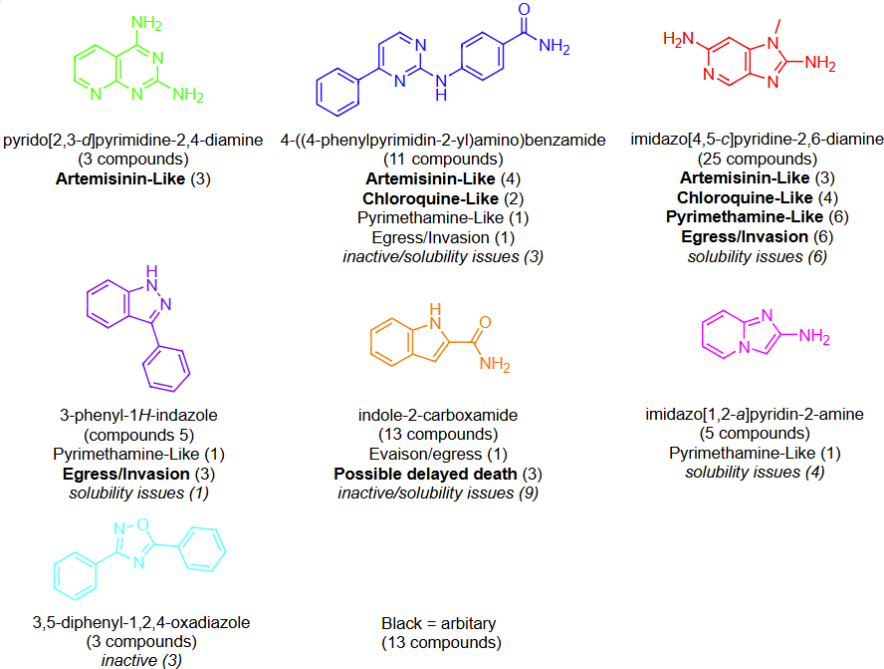
Structural analysis of ZND analogs from the Global Health Priority Box and their correlation with the initial speed of action. Compound numbers from each structural class and speed of action category are listed in the parentheses. Structural classes are color coded for ease of visualization in Table S1.

The ZND section of the Global Health Priority Box is a valuable resource for this study as it contains clusters of structurally related analogs. Out of 80 compounds, 65 analogs could be classified into 7 structural clusters with only 13 compounds not falling within a structural class (denoted as “arbitrary”) (Fig. 5). The structural classes in the ZND section act as a reasonable training set to determine the alignment of a structural class with a speed of action classification. Theoretically, compounds that belong to the same structural class should have the same mechanism of action and therefore the same speed of action.

The structural analysis revealed that pyrido[2,3-d]pyrimidine-2,4-diamine class represented by three compounds was all fast acting (artemisinin like). From the 4-((4-phenylpyrimidin-2-yl)amino)benzamide class, 6/11 were classified as fast-to-intermediate acting (artemisinin-to-chloroquine like), with three compounds classified as inactive or excluded due to solubility issues. The 3-phenyl-1H-indazole class (3/5) was classified as likely egress/invasion inhibitors, and the indole-2-carboxamide class was classified as delayed death—although most of these analogs were either inactive or had solubility issues. The imidazo[4,5 c]pyridine-2,6-diamine structural class was the largest, with 25 derivatives. This class had analogs ranging from fast artemisinin like (three compounds), intermediate chloroquine like (four compounds), slow-acting pyrimethamine like (six compounds), and egress/invasion categories (six compounds) with six compounds excluded due to solubility issues. The imidazo[4,5 c]pyridine-2,6-diamine compound class has been developed for their anti-malarial activity (patent applications, US11344554 and US10736899), and although the mechanism of action is unknown, it is likely they are kinase inhibitors based on their chemical structure. It is possible that within this structural class, small structural modifications may alter the type or number of kinases inhibited, resulting in the broad range of killing rates observed (61). And finally, the 3,5-diphenyl-1,2,4-oxadiazole and imidazo[1,2 a]pyridine-2-amine classes were either inactive or had solubility issues so were not classified. Overall, with the exception of the imidazo[4,5 c]pyridine-2,6-diamines, the analysis revealed analogs from within a structural class correlated with a speed of action classification.

**Fig 6:**
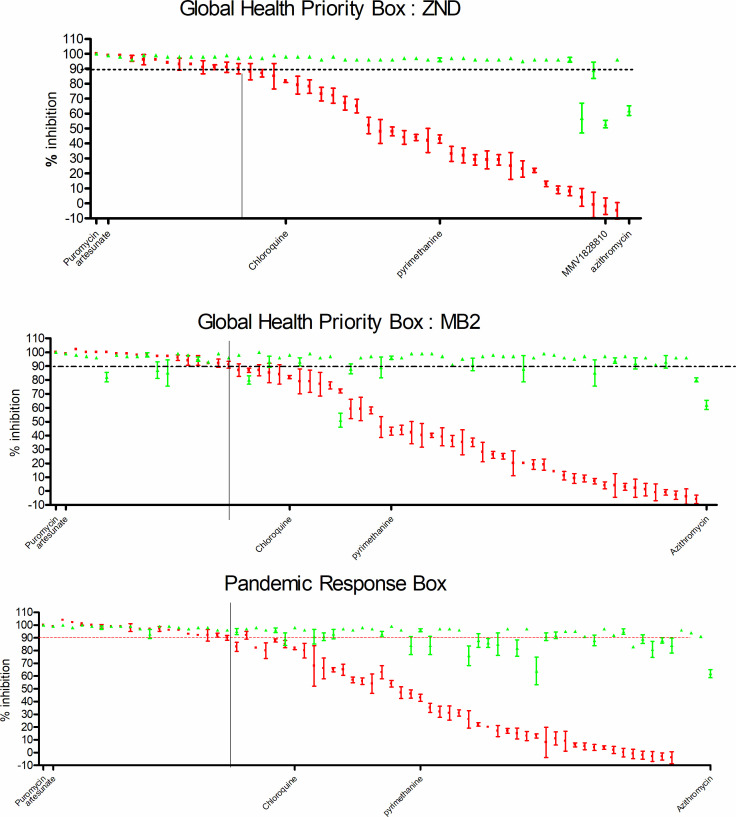
Primary screening of Global Health Priority Box: ZND and MB2 subsets and Pandemic Response Box. The Emax (percent inhibition) is displayed on the Y-axis and the compound and reference identifiers on the X-axis. Red is the mean plus SD of four individual data points from the 38-h SMIA and green for the 120-h assay data. Compounds with Emax (percent inhibition) above 90% for the SMIA (red data points) are classified as fast ring-stage actives, and those under this value are represented by the horizontal line as slower acting (complete data sets provided in Table S1).

In summary, of the 80 compounds, 16 were inactive and a further 21 designated as active but with solubility issues. Of the remaining 43 compounds, 11 were designated as artemisinin like (7 from malaria screening, 2 from tuberculosis, and 1 each from Chagas and leishmania), 6 chloroquine like (all malaria), 9 pyrimethamine like (8/9 malaria), 13 as potential egress/invasion inhibitors (9 malaria, and 1 each of tuberculosis, human African trypanosomiasis, filariasis, and cryptosporidiosis), and 4 with a possible delayed death effect with 3 of the 4 from the TB subset of compounds (Fig. 6; Global Health Priority Box: ZND).

#### Malaria Box 2

All 80 compounds demonstrated activity; however, 20 compounds had standard deviation values of >20% inhibition associated with solubility issues and were thus excluded from the classification. Of the classified compounds (60), 16 were designated as artemisinin like, 10 chloroquine like, 12 pyrimethamine like, and 22 as invasion/egress active (Fig. 6B).

#### Pandemic Response Box

From the total of 400 compounds, 77 were deemed as active, 18 as artemisinin like, 6 chloroquine like, 15 pyrimethamine like, 24 as invasion/egress/low actives, and 14 were excluded from speed of action classification due to solubility issues. Of the 24 compounds classified as slow-acting invasion/egress, 8 have potential activity through DHFR [MMV021759 (TCMDC-131943), MMV1580849, MMV1579846, MMV1581549, MMV000028 (trimethoprim), MMV1579776, MMV1613563, and MMV1580842] (Fig. 6C).

### Retest IC_50_ determination at 38 (SMIA), 65, and 120 h

To validate this approach, compounds above a 90% simulated Emax in the SMIA were selected from each compound subset from the Global Health Priority Box (11 ZND, 16 MB2) for retest in the complete IC_50_ version of this speed of action and stage specificity screening approach. Seven slow-acting compounds from the MB2 compound set were also taken forward to confirm the separation of slower-acting compounds in this testing format. The data obtained for ZND and MB2 compound subsets are presented in Table 3.

**TABLE 3:** Global Health Priority Box: ZND and MB2 IC_50_ and speed of action confirmation^*a*^

	Disease	Speed of action	38-h SMIA			65 h			120 h				
			IC_50_ (nM)	SD	Emax	IC_50_ (nM)	SD	Emax	IC_50_ (nM)	SD	Emax	38/65 h	38/120 h
GHPB:ZND													
MMV1593278	Chagas	Fast ring	1,425	673	103	1,141	377	91	723	9.3	96	1.2	2.0
MMV1828776	TB	Fast ring	1,540	170	99	1,233	101	101	773	77	99	1.2	2.0
MMV1828058	TB	Fast ring	817	16	96	1,328	301	98	827	48	99	0.6	1.0
MMV692630	Malaria	Fast ring	905	85	92	1,148	359	92	675	16	99	0.8	1.3
MMV1579936	Leish	Fast ring	1,045	4.5	90	1,180	198	96	1,140	23	99	0.9	0.9
MMV692115	Malaria	Fast ring	668	74	90	734	93	94	686	39	98	0.9	1.0
MMV689461	Malaria	Fast ring	1,775	66	89	1,430	54	97	1,488	109	99	1.2	1.2
MMV692857	Malaria	Fast ring	185	19	88	227	36	95	215	20	99	0.8	0.9
MMV1542798	Malaria	Fast ring	273	56	88	271	15	94	271	15	94	1.0	1.0
MMV1795508	Malaria	Fast ring	121	25	80	116	20	94	101	1.5	98	1.0	1.2
MMV893278	Malaria	Fast ring	98%			833	25	91	722	587–887 CI	99	0.0	0.0
GHPB:MB2													
MMV006597	Malaria	Fast ring	1,342	354	105	2,210	211	94	1,430	67	99	0.6	0.9
MMV1841740	Malaria	Fast ring	238.6	0.4	100	314	8.6	99	355	26	99	0.8	0.7
MMV674132	Malaria	Fast ring	596	27	100	477	25	94	359	3.8	98	1.2	1.7
MMV1796046	Malaria	Fast ring	2,092	298	100	4,157	306	99	3,207	24	99	0.5	0.7
MMV1845852	Malaria	Fast ring	106	3.6	99	201	18	101	101	3	98	0.5	1.0
MMV020196	Malaria	Fast ring	880.7	51	99	968	51	99	1,132	52	100	0.9	0.8
MMV006430	Malaria	Fast ring	1,799	126	99	2,602	729	85	1,691	61	99	0.7	1.1
MMV020335	Malaria	Fast ring	747.4	43	98	776	46	99	888	30	100	1.0	0.8
MMV1804559	Malaria	Fast ring	492.3	28	97	494	12	99	581	65	99	1.0	0.8
MMV1791425	Malaria	Fast ring	1,209	44	97	1,794	196	97	1,641	73	97	0.7	0.7
MMV020445	Malaria	Fast ring	328	53	95	511	27	98	549	2.6	99	0.6	0.6
MMV978042	Malaria	Fast ring	680.8	29	93	791	130	98	742	93	99	0.9	0.9
MMV014853	Malaria	Fast ring	1,364	68	93	1,520	214	96	1,387	57	99	0.9	1.0
MMV024638	Malaria	Fast ring	192.3	17	92	198	3.1	100	103	22	93	1.0	1.9
MMV006931	Malaria	Fast ring	1,209	223	91	1,442	45	98	1,534	62	99	0.8	0.8
MMV007300	Malaria	Slow	129.1	10	82	122	16	94	134	12	99	1.1	1.0
MMV026953	Malaria	Slow	63	4.7	79	78	2.6	93	59	3.3	97	0.8	1.1
MMV020508	Malaria	Slow	829.2	69	79	990	101	94	1,144	180	99	0.8	0.7
MMV1542706	Malaria	Slow	10.7	1.8	71	28	5.7	91	19	4.1	99	0.4	0.6
MMV1262756	Malaria	Slow	1,303	421	59	756	559	39	311	31	83	1.7	4.2
MMV019053	Malaria	Slow	574.8	9.4	41	637	17	81	655	73	95	0.9	0.9
MMV007282	Malaria	Fast ring	IA						IA				

^*a*^ IC_50_ and Emax data are presented (*n* = 2 biological replicates) and tested in 16-point dose response in duplicate point. The IC_50_-fold shift at 65 and 120 h is presented in the last two columns. The three shaded compounds at retest IC_50_ evaluation were reclassified as chloroquine like.

### Global Health Priority Box: ZND

Of the 11 fast ring-stage actives from the primary abridged screening protocol, their activity distribution recorded in the MMV supporting information against the following diseases was seven malaria, two tuberculosis, one Chagas, and one leishmaniasis. Following IC_50_ retest, 63% (7/11) of the compounds reconfirmed as fast ring-stage artemisinin like (MMV1593278, MMV1828776, MMV1828058, MMV692630, MMV1579936, MMV692115, and MMV689461) and consisted of 3 from the pyrido[2,3-d]pyrimidine-2,4-diamine chemotypes (MMV1828776, MMV1828058, and MMV1579936) and 3 4-((4-phenylpyrimidin-2-yl)amino)benzamide compounds (MMV692630, MMV692115, and MMV689461). Three imidazo[4,5 c]pyridine-2,6-diamine compounds (MMV692857, MMV1542789, and MMV1795508) were reclassified as chloroquine like based on the retest data. This evaluation identified two chemotypes (3 pyrido[2,3-d]pyrimidine-2,4-diamine and 4-((4-phenylpyrimidin-2-yl)amino)benzamide) with fast ring-stage activity. However, the activity is only within the 600–1,500 nM range, with those identified as chloroquine like demonstrating a greater level of activity (100–280 nM) (Table S1).

### Global Health Priority Box: MB2

Sixteen compounds were selected as fast ring-stage actives and seven slow acting, with 93% of the 16 fast-acting compounds (MMV674132, MMV1791425, MMV1845852, MMV007282, MMV020196, MMV006597, MMV1804559, MMV1841740, MMV024638, MMV020335, MMV006430, MMV1796046, MMV020445, MMV978042, MMV006931, and MMV014853) confirmed as fast ring-stage active (artemisinin like). Of the seven slower-acting compounds identified as such at primary testing, 6/7 (86%) confirmed as slow acting (MMV026953, MMV1262756, MMV019053, MMV1542706, MMV007300, and MMV020508), with one compound (MMV019177) having insufficient activity to generate an IC_50_ at any incubation time point (Table 3). Data for MMV1263756 support the differentiation of fast and slow compound activities based on its potential mechanisms of action as determined by resistance pooling and testing against a panel of resistant strains. This compound is flagged as potentially having some activity through DHODH in the Global Health Priority Box supporting information. In this study, MMV1263756 has a trophozoite stage of arrest and IC_50_ value fold shifts of 1.7 (65 h) and 4.2 at 120 h like atovaquone and DSM1. Thus, the data demonstrated that the abridged screening version could distinguish fast from slow-acting compounds, with lag-phase activity identified in follow-up IC_50_-fold shift evaluation. In summary, 16 fast ring-stage active compounds were identified from this screening campaign with inhibitory activities ranging from 100 to 3,000 nM IC_50_ values.

Of note, reanalysis of the 10 µM data for the SMIA for the MB2 compounds, using a percent inhibition cutoff >90% identified the same fast active ring-stage compounds obtained from either the abridged or full IC_50_-fold shift approach. Thus, demonstrating the feasibility of the schizont maturation assay as a means of screening in high-throughput format to identify compounds with a high likelihood of having fast ring-stage activity against any *Plasmodium falciparum* strain or clinical isolate culturable *in vitro*.

### Pandemic Response Box

Of the 18 fast ring-stage active compounds identified at primary screening in the abridged screening approach [MMV1634391 (MGB-BP3, Strathclyde Minor Groove Binder antibiotic), MMV1578884 (CRS-3123), MMV637413 (fludarabine), MMV639951 (Everolimus), MMV1006203, MMV010036 (Panobinostat), MMV002722 (Miconazole), MMV163449, MMV1578574 (Eravacycline), MMV1634492 (Eberconazole), MMV1580482 (URMC-099-C), MMV1557865 (Brinapant), MMV1578890, MMV000725, MMV394033, MMV1580483 (AZD-0156), MMV687273 (AQ109), and MMV011565 (GNF-Pf-117)], 6 were selected for retest in the complete IC_50_-fold shift approach. MMV1634391 (MGB-BP3) and MMV1580482 (URMC-099-C) confirmed fast ring-stage activity. MMV1578884 (CRS-3123), MMV010036 (Panobinostat), and MMV15578561 (Brinapant) demonstrated a potential lag with IC_50_-fold shifts of >2.4 but only after the second cycle of proliferation. A new stock of everolimus (MMV639951) did not demonstrate sufficient activity to generate IC_50_ values at any time point. MMV1578560 (OSU-03012) and MMV1580853 (BPH-1358) from the six chloroquine-like subsets [MMV1782353 (compound K), MMV1578560 (OSU-03012), MMV1580853 (BPH-1358), MMV000008 (chloroquine), MMV1578561, and MMV1578561] were further evaluated. At retest, MMV1578560 (OSU-03012) was reclassified as slow acting with an Emax and IC_50_ value obtainable only at the 120-h evaluation and MMV1580853 (BPH-1358) to be fast ring-stage active based on Emax (94%), but the large IC_50_-fold shift values (4.6 and 10.5 at 65 and 120 h, respectively) reduced the classification of this compound to be slow acting with a lag phase in its development.

Of the 15 pyrimethamine-like compounds [MMV1580496 (triapine), MMV009948 (Imatinib), MMV687800 (clofazimine), MMV637528 (Itraconazole), MMV1593540, MMV1634399, MMV642550, MMV1580488 (ML 324), MMV002731 (ciclopirox), MMV1582497, MMV1580844 (DHFR potential by structure), MMV658803 (Tipifarnib), MMV1580492 (Ozanimod), MMV1581554 (NSC731130), and MMV565773 (NSC84094)], 5 were selected for further evaluation. MMV1580496 (triapine), MMV687800 (clofazimine), MMV1580488 (ML324), MMV658803 (tiparnib), and MMV1580492 (Ozanimod) confirmed as slow acting. Of the 24 slow-acting invasion/egress-classified compounds, 3 [MMV396785 (Alexidine), MMV1634402 (Brilacidin), and tafenoquine (MMV000043] were taken forward for further evaluation. Alexidine (MMV396785) demonstrated a 47% Emax at 38 h, and IC_50_-fold shifts of 2.3 and 6.4 for 65- and 120-h evaluations, respectively, suggestive of a role in mitochondrial deactivation like that of atovaquone and DSM1. Brilacidin confirmed as a slow-acting compound, with IC_50_ values only obtainable after one cycle of proliferation. Tafenoquine (MMV000043) demonstrated autofluorescence interference in this assay format, particularly at the higher concentrations, which was sufficient to impact on the detection of parasites at 38 and 65 h of incubation, thus resulting in large negative inhibition values. Ten other compounds also demonstrated large negative percent inhibition values, four with positive activity at 120 h (MMV019724, MMV1633968, MMV1593537, and MMV1593541) and six identified as inactive at 120 h (MMV1580498, MMV1782113, MMV1782107, MMV1580799, MMV099714, and MMV1580846). The four compounds (but not tafenoquine) active at 120 h were not included in the classification analysis due to the potential autofluorescent interference in the homogeneous assay format of this screening assay approach. All compound activity data including IC_50_ values are provided in Table S1.

These data demonstrated that the abridged version of the speed of action screening approach can differentiate slow-acting compounds from potentially fast ring-stage active compounds within a malaria-naïve compound set. Further evaluation of actives from the primary abridged testing in the IC_50_-fold shift complete screening format could further classify fast ring-stage active compounds into those which have an extended lag phase, such as atovaquone (>2.5-fold IC_50_ shift at 65 h), from those with an immediate fast action against ring-stage asexual *Plasmodium falciparum* parasites, such as artesunate.

## DISCUSSION

We present a screening approach capable of identifying anti-malarials, their speed of action, and the parasite stage at which growth is arrested in high-throughput format.

Utilizing a set of well-characterized reference anti-malarial compounds, two compound sets from the Global Health Priority Box, namely the ZND and MB2 sets consisting of 80 compounds each, and 400 compounds from the malaria-naïve Pandemic Response Box, we demonstrated the utility of this screening approach in two distinct formats. First, a full IC_50_-shift approach using all three time points, 38 h SMIA, 65 h single, and 120 h two cycles of proliferation (Fig. 1), a second abridged format utilizing only two moderately high compound concentrations (10 and 5 µM) and only two time points, SMIA (38 h) and two cycles of proliferation (120 h). The data for the malaria set belonging to the Pathogen Box are particularly informative, as this compound collection has been extensively investigated (28, 37, 41–45), providing published data for comparison to be drawn and, in many cases, proposed mechanisms of action and speed of kill data.

Published anti-malarial data for the Global Health Priority Box ZND and MB2 compound sets are limited, with most of the information provided by MMV as supporting information (included in Table S1). We believe data presented here to be the first published data for anti-malarial activities of these compound collections (ZND and MB2), while activities for other pathogens, such as helminths (62) and causative agents of Eumycetoma (63), have been published. MMV804559 identified as active against *Madurella mycetomatis* (63) is identified as fast ring stage active in this study. Our evaluation of the ZND compound set indicated a clustering of chemotypes, which aligned in general with the speed of action and parasite stage of arrest (Fig. 5). Interestingly, the imidazo[4,5 c]pyridine-2,6-diamine compound class, expected to be kinase inhibitors, demonstrated a range of activity classifications in this study, which may be related to kinase specificity, an occurrence that has previously been reported (61).

The MB2 compounds are those with no apparent parasite resistance and therefore of great importance for malaria drug discovery. Using the abridged two doses, two time points format, 16 fast ring-stage active compounds were identified and confirmed using the complete IC_50_-fold shift approach. Reanalysis of the MB2 data set from the SMIA using only a single 10 or 5 µM screening dose identified the same fast ring-stage active compounds from the two-dose, two-time point abridged screening format (these being MMV674132, MMV1791425, MMV1845852, MMV007282, MMV020196, MMV006597, MMV1804559, MMV1841740, MMV024638, MMV020335, MMV006430, MMV1796046, MMV020445, MMV978042, MMV006931, and MMV014853), thus demonstrating the capability of the SMIA to identify and prioritize fast ring-stage active compounds at a single high screening dose from a malaria-focused compound library. As fast ring-stage active compounds are potentially more effective clinically (14) and less prone to parasite resistance (13), this capability to identify and prioritize fast ring-stage activity is of exceptional value in early malaria drug discovery.

Testing of the Pandemic Response Box yielded mixed results regarding speed of action and stage specificity in the abridged screening format. Of five compounds identified to be slow acting [MMV687800 (clofazimine), MMV1580496 (triapine), MMV1580488 (ML324), MMV658803 (Tipifarnib) and MMV1580492 (Ozanimod), all five confirmed slow activity in the full IC_50_-fold shift approach. In the case of 6 fast ring stage actives taken for full IC_50_-fold shift testing, only two [MMV1634391 (MGB-BP3) and MMV1580482 (URMC-099-C)] confirmed as fast-acting ring-stag- specific compounds, with MMV1578884 (CRS-3123), MMV010036 (panobinostat), and MMV1557856 (birinapant) all demonstrating a lag phase between replication cycles and therefore slow activities. A single fast ring-stage active compound MMV639951 (everolimus) demonstrated a slow speed of action at retest in the full IC_50_-fold shift approach.

This new compound screening approach provided flexibility at all stages of early drug discovery from single-dose SMIA HTS to identify fast ring-stage active compounds, an abridged version to identify and classify activities into fast- and slow-acting compounds with an indication of stage specificity. Plus, a full IC_50_-shift format that can determine not only the speed of action and stage specificity but also can identify changes in both parameters previously reported during lead optimization, where speed of action and molecular target switching were identified (61).

The application of the original PRR assay for evaluating the impact of two or three drug combinations on speed of action and parasite clearance times *in vitro* is gaining momentum for malaria drug discovery (64). Based on the ability of this new high-content initial speed of action and stage of parasite growth arrest screening approach, and its correlation with the basic output of the PRR, it is expected that this approach will also enable the evaluation of the effect of drug combinations on the initial speed of action and stage specificity of these drug combinations. With its much higher-throughput capacity, this approach could fast track drug combination activity far earlier in the drug discovery process, thereby further derisking drug discovery programs and prioritizing those with the greatest biological potential.

## MATERIALS AND METHODS

### *Plasmodium falciparum in vitro* culture

*Plasmodium falciparum* 3D7 (MRA-102), obtained from BEI resources, was cultured in complete culture medium comprising RPMI-1640 (R8758 Merck) supplemented with 50 mM HEPES (H0887-100ML Merck), 50 µg/mL hypoxanthine, 2.5 mg/mL Albumax II (11021045 Thermo Fisher), and 5% human serum (H4522-100ML, Merck). Parasitemia (P) was maintained at a maximum of 1%—2% trophozoites and 5%—8% ring stages at 5% hematocrit (H) in human O-positive RBC. All incubations were in Tri-gas incubators set to 5% CO_2_and 5% O_2_ at 37°C. The parasite cultures were synchronized to ring-stage parasites by a single sorbitol treatment (29, 65) at every second cycle of proliferation in preparation for performing the isolation of 0–3-h ring-stage parasites.

### Isolation of 0–3-h ring-stage parasites

The protocol used to isolate 0–3-h ring-stage parasites is described in depth elsewhere (29) with some minor changes. Briefly, schizonts were isolated from 50 mL of schizont containing *Pf*3D7 culture using a CS MACs column (130–041-305 Miltenyi Biotec Australia) and 0.5 mL of compacted RBC added to the isolated schizonts. The culture was incubated using the conditions for *in vitro* culture and employed for all parasite handling in this study. After 3 h of incubation in 5% CO_2_ and 5% O_2_ at 37°C, the culture was treated with sorbitol (1/2 the volume of the culture volume), lysing any remaining schizonts and resulting in 0–3-h ring-stage parasites.

### Compound handling

#### Medicines for Malaria Venture Pathogen Box anti-malarial compounds (125), Global Priority Box (160), and Pandemic Response Box compounds (400)

Ten microliters of 10 mM compound stocks in 96-well plates were received from MMV (Geneva, Switzerland). The Pathogen Box compounds (125) were diluted to 5 mM in 100% DMSO, transferred to 384-well polypropylene plates, and serially diluted in 100% DMSO from 5 mM in a 16-dose response, three concentrations per log in duplicate point. The stock compound plates were then diluted in water (1/25) before 5 µL transferred into PDL (Poly-D-Lysine)-coated Cell carrier Ultra imaging plates (Revvity) to provide final screening concentrations of 20 µM down to 0.2 nM.

The Global Health Priority Box (160 compounds in total) and Pandemic Response Box (400 compounds) were diluted to 2.5 mM in 100% DMSO and transferred into 384-well polypropylene plates. As for the Pathogen Box compounds, a 1/25 dilution in water was performed (final assay concentration of 10 µM), followed by a further 1/2 dilution in 4% DMSO to provide the second final screening dose (5 µM). Five microliters of each dilution were transferred to PDL-coated Cell carrier Ultra imaging plates (in duplicate).

#### Reference compound preparation

Reference compounds were prepared as 10 mM stocks in 100% DMSO. Further dilutions in 100% DMSO were made to provide appropriate dose-response curves for the compounds with a diverse range of activities. The dose-response curves were diluted 1 in 25 in water and 5 µL transferred into PDL-coated Cellcarrier ultra imaging plates. Puromycin, the nonspecific, protein synthesis inhibitor active at all stages of *Pf* asexual development (29), was used as the positive 100% inhibition control in conjunction with 0.4% DMSO (negative control) to normalize all data for percent inhibition calculation.

### Pandemic Response Box retest from commercially available solids

Solid samples of compounds were purchased from MedchemExpress and Saphire Biosciences. Compounds were solubilized to 10 mM in 100% DMSO and processed as per reference compounds for testing in the SMIA (38 h), 65- and 120-h assays.

#### Assay culture preparation

The 0–3-h ring-stage parasite culture obtained by magnetic column isolation was adjusted to 2% P at 0.3% hematocrit in complete culture medium. Forty-five microliters of culture were then dispensed into the compound containing assay plates, which were lidded and incubated for 38, 65, or 120 h under the conditions described in for *in vitro* parasite culture conditions.

#### DAPI staining

After 38, 65, or 120 h of incubation, DAPI (1 mg/mL stock in dimethylformamide) was diluted to 1 µg/mL in phosphate-buffered salin supplemented with 0.01% Triton X-100 and 0.1 mg/mL saponin (Merck) made fresh on the day. Thirty microliters of the DAPI were added to all wells in 384-well plates using a Biomek FX liquid handling instrument. The plates were incubated in the dark for at least 12 h before imaging on the Phenix High Content Imaging System (Revity).

### Imaging and analysis

The principles of the image acquisition and analysis are described elsewhere (21). The assay plates in this study were imaged using the Phenix High Content Imaging System (Revitty.com), utilizing instrument setting for DAPI (405 nm excitation, 435–480 nm emission with a 120-m exposure at 100% power and a read height of −8.0 µm), with three images per well acquired using a 20× water objective (NA 1.0). The images were analyzed using Harmony software (Part Number: HH17000016 version 4.8) and spot detection building blocks for image analysis (“Find spots” method C, with the following parameters, ≤2.21 µm spot radius and >0.23 contrast), which was manually taught to provide the optimized script parameters. The analysis output was total classified spots (parasites) normalized to puromycin and 0.4% DMSO controls to generate percent inhibition values.

### Development of the schizont maturation inhibition assay

#### Validation of the detection of parasite development utilizing DAPI staining and image analysis

Forty-five microliters of highly synchronous ring-stage parasites (0–3 h post RBC infection) at 2% P and 0.3% H were added to 384-well PDL-coated Cell carrier Ultra imaging plates (Revvity). The plates were incubated, and puromycin at 10 µM was added to 32 wells at 5, 10, 15, 20, 25, 32, and 35 h post RBC invasion. After 65 h of total incubation, all the puromycin-treated wells were stained with DAPI staining reagent and imaged using a Phenix High Content Imaging System .

### Validation of the schizont maturation inhibition assay to classify reference anti-malarial drugs as fast or slow acting, with indication of parasite stage of arrest

Forty-five microliters of 0–3-h ring-stage parasite culture at 2% P and 0.3% H (the same as the asexual HTS conditions) were added to diluted compound containing wells, including control wells containing 4% DMSO and 50 µM puromycin (final assay concentrations of 0.4% DMSO and 5 µM puromycin). Two-milliliter samples of the assay ready culture were also exposed to 10 µM of each compound tested for 38 h.

The 384-well assay plates were then incubated for 38 h at 5% O_2_, 5% CO_2_ at 37°C in a Trigas incubator with 60% relative humidity. After 38 h, the plates were stained by the addition of 30 µL of the DAPI using a Biomek FX liquid handler. The plates were left at RT in the dark for at least 12 h prior to imaging on the Phenix High Content Imaging System. At 38 h, the 2-mL compound-treated parasite cultures were centrifuged, and small volumes of the pelleted cells smeared onto microscope slides which were dried, fixed with methanol, and then stained with Giemsa reagent for 10 minutes (Merck 48900–500ML-F). Images of the parasites were acquired using 100× oil immersion light microscope.

### Data normalization and determination of IC_50_ values

The classified parasite number obtained from the automated Harmony image analysis software was normalized by calculating percent inhibition in relation to the DMSO and puromycin control data [percent inhibition calculation = 100 − (test − puromycin)/(DMSO − puromycin) × 100].

The percent inhibition for each concentration was plotted in Prizm 4.1 using nonlinear regression fit, Sigmoidal dose response (variable slope), with no constraints applied to either top or bottom of the curve fit.

## SUPPLEMENTAL MATERIAL

Supplemental legend aac.00746-24-s0001.docx Legend for Table S1.

Table S1 aac.00746-24-s0002.xlsx Complete activity data summary.

ASM does not own the copyrights to Supplemental Material that may be linked to, or accessed through, an article. The authors have granted ASM a non-exclusive, world-wide license to publish the Supplemental Material files. Please contact the corresponding author directly for reuse.
