# Supplementary material for: An adaptable, fit-for-purpose screening approach with high-throughput capability to determine speed of action and stage specificity of anti-malarial compounds
Source: Antimicrob Agents Chemother. 2024 Sep 12;68(10):e00746-24. doi: 10.1128/aac.00746-24 (PMC11459970; doi:10.1128/aac.00746-24)
Supplement: Supplemental legend — Legend for Table S1. [file aac.00746-24-s0001.docx]

Table S1.

Complete activity data and supporting information for MMV Pathogen Box malaria set, MMV Global Health Priority Box (ZND and MB2) and MMV Pandemic Response Box.
